# Isolated Unilateral Infiltrative Optic Neuropathy in a Patient with Breast Cancer

**DOI:** 10.4274/tjo.galenos.2018.24478

**Published:** 2019-06-27

**Authors:** Kaveh Abri Aghdam, Amin Zand, Mostafa Soltan Sanjari

**Affiliations:** 1 Eye Research Center, The Five Senses Institute, Rassoul Akram Hospital, Iran University of Medical Sciences, Tehran, Iran

**Keywords:** Optic nerve head infiltration, breast cancer, ocular metastasis

## Abstract

Metastasis to the optic nerve is very rare. We report a case of metastatic breast cancer to the optic nerve head without the involvement of other ocular or orbital structures. The patient, a 39-year-old female who had been previously treated for breast cancer, reported a gradually progressive decrement in visual acuity of the right eye during the past two months. Fundus examination of the affected eye revealed swelling of the optic disc which was infiltrated by a yellowish mass. Further evaluation using optical coherence tomography and fluorescein angiography showed optic disc swelling. Magnetic resonance imaging revealed no pathologic findings. With a diagnosis of unilateral infiltrative optic neuropathy, we referred the patient to an oncologist for further evaluation.

## Introduction

Ocular metastasis is rare and metastasis to the optic nerve is even rarer. In a case series study, Shields et al.^1^ reported metastases to the optic disc in 4.5% of patients with ocular metastases. In another study on patients with ocular metastasis, orbital metastasis, or both, Ferry and Font^2^ reported that 1.3% involved metastases limited to the optic nerve, and metastatic breast cancer was seen in only 0.4%. We report a case of metastatic breast cancer to the optic nerve head which was unilateral. The diagnosis was based upon the previous history of breast cancer, optic disc examination in the affected eye, and imaging results.

## Case Report

The patient was a 39-year-old female who had experienced a gradually progressive decrement in visual acuity of the right eye during the past 2 months. Her medical history indicated that she had been treated for breast carcinoma, which had been originally diagnosed in her right breast 6 years ago, with no signs of metastases. Histopathological evaluation confirmed invasive ductal adenocarcinoma of the breast. She had been since treated by mastectomy and adjuvant chemotherapy with docetaxel until 3 years ago when her treatment with oral tamoxifen was begun. The treatment limited the neoplastic process and there were no clinical or radiological signs of progressive disease during these years.

The patient had no significant medical history. She was taking tamoxifen. She had no history of alcohol or tobacco use and there was no environmental toxic exposure. Her family history was negative for breast cancer and other diseases.

**Figure 1:**
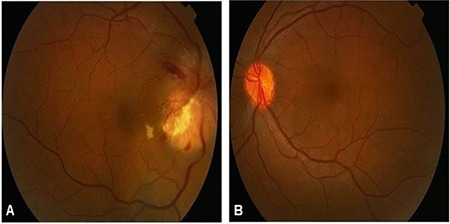
Fundus photography of both eyes. (A) Optic disc swelling with obscuration of blood vessels and peripapillary flame-shaped hemorrhages. A large yellowish infiltrative mass, with disruption of the architecture of the optic disc is noticeable. (B) The left eye seems normal

**Figure 2:**
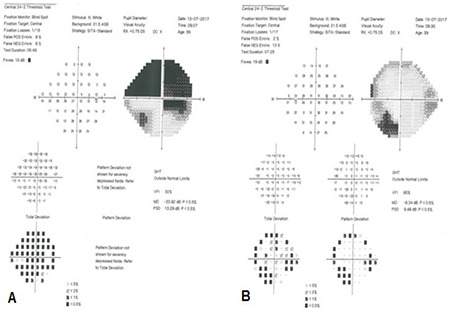
Humphrey visual field testing in both eyes. (A) The right eye shows an altitudinal defect with enlargement of the blind spot. (B) The left eye shows a non-specific arcuate scotoma

**Figure 3:**
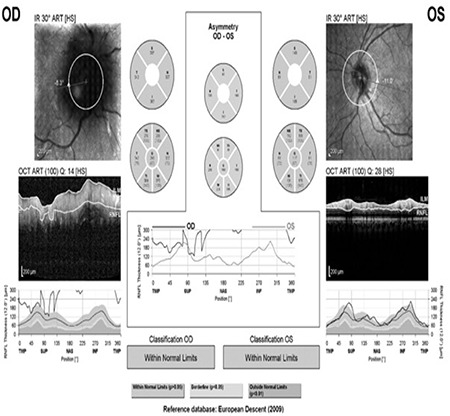
Peripapillary optical coherence tomography (PP-OCT) of both eyes. The right eye shows significant thickening of the retinal nerve fiber layer in all four quadrants due to optic disc swelling and an infiltrative mass. PP-OCT of the left eye is normal

**Figure 4:**
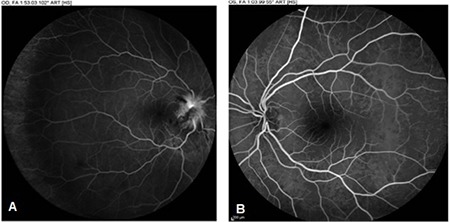
Fluorescein angiography of both eyes, mid phase. (A) Hyperfluorescent mass on the right optic disc with no evidence of leakage along with peripapillary hypofluorescent areas compatible with blocking effect from flamed-shape hemorrhages. (B) Fluorescein angiography of the left eye seems normal

**Figure 5:**
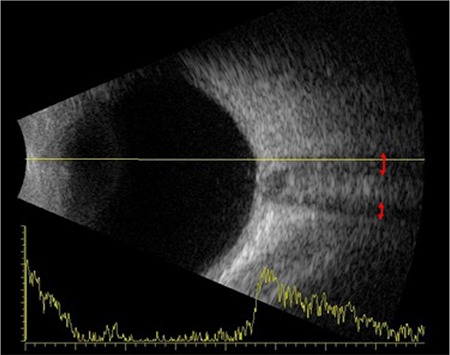
B-scan ultrasonography of the right eye shows abnormally increased optic nerve sheath diameter (red double-headed arrows)

Office examination revealed a best-corrected visual acuity of counting fingers at 2 meters in the right eye and 10/10 in the left eye (by Snellen E chart from six meters). There was a 3^+^ relative afferent papillary defect in the right eye. Extraocular motility was intact in both eyes. Intraocular pressures were within normal limits in both eyes in applanation tonometry. Color plate testing results (by Ishihara’s color plate test) was 1/14 for the right eye and 14/14 for the left eye. Anterior segment examination was unremarkable. Dilated fundus examination of the right eye demonstrated 1^+^ cells in the vitreous, optic disc swelling, obscuration of vessels and infiltration by a large yellowish mass that disrupted the normal structure of the optic disc, and flame-shaped hemorrhages in the peripapillary (PP) region (Figure 1). Fundus examination of the left eye was normal. Humphrey visual field testing in the right eye showed an altitudinal defect with enlarged blind spot (Figure 2). PP optical coherence tomography (OCT) demonstrated significant retinal nerve fiber layer thickening in all four quadrants in the right eye (Figure 3). Fluorescein angiography (FA) of the right eye detected a hyperfluorescent mass on the right optic disc with no sign of leakage, which suggested infiltrative optic neuropathy (Figure 4). Humphrey visual field testing in the left eye revealed a non-specific arcuate scotoma (Figure 2). OCT and FA in the left eye were normal (Figures 3 and 4). B-Scan ultrasonography of right eye revealed slight abnormal increase in right optic nerve sheath diameter (Figure 5). Magnetic resonance imaging (MRI) was unremarkable and intraorbital and intracranial portions of both optic nerves had normal appearance.

According to the patient’s present condition, her past history of breast cancer, optic disc features on fundus examination, and imaging findings, the first diagnosis was infiltrative optic neuropathy of the right eye. The patient was referred to an oncologist for further systemic examination and necessary interventions.

## Discussion

The optic nerve can be infiltrated by primary or secondary tumors and inflammatory processes. The most common secondary tumors that involve the optic nerve are metastatic and locally invasive carcinomas and hematologic malignancies, especially lymphoma and leukemia.^3^ Metastases can reach the optic nerve via the choroid, by vascular dissemination, by invasion from the orbit, and through the central nervous system.^4,5,6^ In patients with infiltrative lesions of the optic nerve, optic disc elevation can be due to swelling and/or infiltrative masses. Consequently, the patient may have impairments in visual acuity, color vision, and visual field in the affected eye(s). When the involvement is unilateral or asymmetric, the patient may have a relative afferent pupillary defect.^3^ In a literature review of 13 patients with breast carcinoma metastasis to the optic nerve, Cherekaev et al.^7^ reported that in the majority of cases (10 of 13), loss of vision was the main symptom. The visual acuity of our patient had diminished progressively, with impaired color vision and a visual field defect in the right eye due to an infiltrative optic neuropathy. She had also a relative afferent pupillary defect due to unilateral involvement of the right eye.

When the metastasis is located in the orbital portion of the optic nerve, the optic disc is usually swollen and a yellow-white infiltrative mass that protrudes from the surface of the nerve can be seen on the optic disc. Tumor cells can sometimes be seen in the vitreous body.^4,8,9,10^ In metastases involving the posterior aspect of the orbital portion of the optic nerve, the optic disc appears normal in the early stages.^3^ In our patient, the right optic disc was swollen with peripapillary flame-shaped hemorrhages and a yellowish infiltrative mass on the disc. Some cells were detected in the vitreous body of the affected eye. PP-OCT also revealed swelling of the affected optic disc. FA findings did not show any evidence of leakage, suggesting infiltrative optic neuropathy.

The most common metastatic tumors to the optic nerve are adenocarcinomas. In females, breast and lung cancers and in males, carcinomas of the lung and intestinal tract are the most common causes.^1,4,11,12,13^ Likewise, carcinomas of the pancreas, stomach, uterus, ovary, kidney, prostate, and larynx can metastasize to the optic nerve.^14,15^ Our patient had a previous history of treatment for breast cancer, which was considered the most probable cause of infiltrative optic neuropathy of the affected eye.

Neuroimaging is crucial in patients suspected of infiltrative optic neuropathy due to cancer. MRI findings include optic nerve enlargement that is diffuse (more common) or in a circumscribed area, associated exudates or hemorrhage, and optic canal involvement in osteophilic metastatic tumors such as prostate carcinoma.^10,11,16,17,18,19^ Our patient had normal orbital and brain MRI.

Most metastatic optic nerve tumors show a variable response to radiotherapy.^17,20^ The prognosis for patients with isolated breast cancer who suffer metastasis to the optic nerve is relatively poor.^21^ We referred our patient to an oncologist for further evaluation and treatment.

In conclusion, most patients with optic nerve metastatic tumors exhibit a known diagnosis of a primary malignancy along with other evidence of metastases. Thus, when a known cancer patient develops optic neuropathy, metastases and infiltration should be suspected as the cause unless proven otherwise.
